# A randomised controlled trial to evaluate the efficacy of a 6 month dietary and physical activity intervention for prostate cancer patients receiving androgen deprivation therapy

**DOI:** 10.1186/1745-6215-11-86

**Published:** 2010-08-12

**Authors:** Farhana Haseen, Liam J Murray, Roisin F O'Neill, Joe M O'Sullivan, Marie M Cantwell

**Affiliations:** 1 Centre for Public Health, Queen's University Belfast, Northern Ireland, UK; 2 Centre for Cancer Research and Cell Biology, Queen's University Belfast, Northern Ireland, UK

## Abstract

**Background:**

Treatment with Androgen Deprivation Therapy (ADT) for prostate cancer is associated with changes in body composition including increased fat and decreased lean mass; increased fatigue, and a reduction in quality of life. No study to date has evaluated the effect of dietary and physical activity modification on the side-effects related to ADT. The aim of this study is to evaluate the efficacy of a 6-month dietary and physical activity intervention for prostate cancer survivors receiving ADT to minimise the changes in body composition, fatigue and quality of life, typically associated with ADT.

**Methods:**

Men are recruited to this study if their treatment plan is to receive ADT for at least 6 months. Men who are randomised to the intervention arm receive a home-based tailored intervention to meet the following guidelines a) ≥ 5 servings vegetables and fruits/day; b) 30%-35% of total energy from fat, and < 10% energy from saturated fat/day; c) 10% of energy from polyunsaturated fat/day; d) limited consumption of processed meats; e) 25-35 gm of fibre/day; f) alcoholic drinks ≤ 28 units/week; g) limited intake of foods high in salt and/or sugar. They are also encouraged to include at least 30 minutes of brisk walking, 5 or more days per week. The primary outcomes are change in body composition, fatigue and quality of life scores. Secondary outcomes include dietary intake, physical activity and perceived stress. Baseline information collected includes: socio-economic status, treatment duration, perceived social support and health status, family history of cancer, co-morbidities, medication and supplement use, barriers to change, and readiness to change their health behaviour. Data for the primary and secondary outcomes will be collected at baseline, 3 and 6 months from 47 intervention and 47 control patients.

**Discussion:**

The results of this study will provide detailed information on diet and physical activity levels in prostate cancer patients treated with ADT and will test the feasibility and efficacy of a diet and physical activity intervention which could provide essential information to develop guidelines for prostate cancer patients to minimise the side effects related to ADT.

**Trial registration:**

ISRCTN trial number ISCRTN75282423

## Background

Prostate cancer is the most common cancer in men in the United Kingdom, with more than 35,000 new cases diagnosed each year [1]. The number of prostate cancer survivors is increasing each year due to advances in cancer detection, treatment and care. Recent statistics (2001-2006) have shown that, in the absence of other competing causes of death, an estimated 77% of those diagnosed with prostate cancer can expect to be alive in 5 years, whereas in 1991-1995 the five-year survival rate was estimated at < 54% [1]. Therefore, as the number of prostate cancer survivors and their length of survival increases, health issues specific to prostate cancer survival are fast emerging as a public health concern.

Treatment of prostate cancer is based on a patient's age, co-morbidity, stage and grade of the prostate tumour and local availability of treatment [2]. Standard therapies for localised prostate cancer include radical prostatectomy, external beam radiotherapy, brachytherapy, often combined with androgen deprivation therapy (ADT). For more advanced prostate cancer ADT is a standard part of the management for the majority of patients and has been conclusively shown to improve survival [3]. Because of increasing evidence of the benefit of ADT in prostate cancer, the number of prescriptions of ADT for treating prostate cancer in the UK increased from 33,000 in 1987 to 470,000 in 2004 [4]. However, treatment with ADT is associated with significant adverse physiological and psychological effects. For example, the reduction in testosterone levels by ADT causes a decrease in lean body mass and muscle strength, a reduction in bone mass and bone mineral density, an increase in fat mass, total body weight and cholesterol levels; a reduction in haemoglobin levels and as a result increased lethargy, which can affect both physical and physiological function [5-7].

A diet and physical activity intervention that could address some of these side effects could be beneficial to prostate cancer patients treated with ADT. Experimental studies examining the role of exercise during ADT treatment for prostate cancer have shown that 10-20 weeks of exercise is an effective way to reduce fatigue and depression, improve quality of life, increase muscular fitness and strength [8-11], and improve physical functional capacity preventing adverse effects on body composition [9]. Indeed 20 weeks of exercise has been shown to preserve lean body mass and prevent an increase in fat mass [11]. Changes in dietary intake at the time of treatment may also help to counteract the changes in body composition associated with ADT. Several randomised controlled trials (RCTs) of diet and physical activity interventions in prostate cancer patients have begun in recent years and include the FRESH START trial, Project LEAD, and RENEW. However the men recruited within these studies have received all types of treatment modalities [12-14]. Therefore, the impact of a diet and physical activity intervention on the side-effects related to ADT, a group that experiences major changes in body composition, has not yet been discussed in any of these studies.

To date very little information is known about dietary intake and physical activity levels of prostate cancer patients in the UK. In general however, cancer patients are highly motivated to seek information about food choices, physical activity, dietary supplement use, and complementary nutritional therapies in an attempt to improve their response to treatment, speed-up recovery rates, reduce the risk of cancer recurrence, and/or to improve their quality of life [15]. The American Cancer Society has recently published nutrition and lifestyle guidelines for cancer survivors [16] although the impact of these guidelines on outcomes relevant to prostate cancer patients treated with ADT are unknown. In addition, for long-term prostate cancer survivors, an appropriate weight, a healthy diet, and a physically active lifestyle aimed at preventing recurrence, second primary cancers, and other chronic diseases should be a priority. Therefore, we have developed an intervention aimed at encouraging patients treated with ADT to meet healthy eating guidelines and achieve at least 30 minutes of brisk walking at least 5 days per week to reduce treatment related side-effects and improve overall quality of life.

### Objectives

#### Primary objective

The study is intended to evaluate the efficacy of a 6 month dietary and physical activity intervention for prostate cancer survivors receiving ADT. The outcomes of interest are body composition, fatigue and quality of life.

#### Specific objectives

We will also describe the nutritional status, dietary intake and physical activity level among prostate cancer survivors in Northern Ireland. The effect of the intervention on dietary intake, level of physical activity, quality of life, fatigue, and psychological stress will be assessed by comparing the intervention group with the controls. Moreover the stages of readiness to change lifestyle behaviours in prostate cancer survivors after diagnosis and during ADT will be measured using the transtheoretical model.

#### Hypothesis

We hypothesize that a diet and physical activity intervention will prevent or reduce weight gain, and minimize the increase in body fat mass typically found in patients treated with ADT in the intervention group compared with the controls. We also hypothesize that the intervention patients will experience less fatigue and will have a better quality of life score compared with the controls.

## Method

### Design

**Figure 1:**
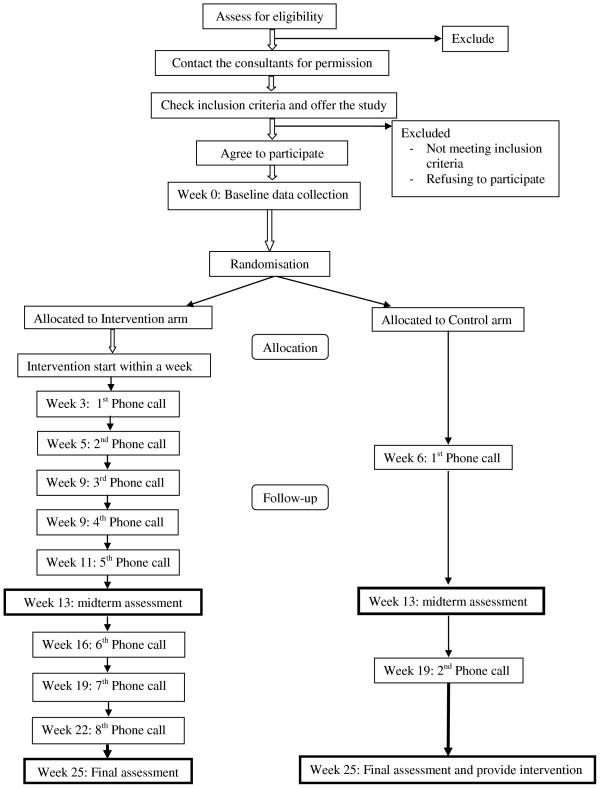
**Study Design in flow diagram**. Recruitment, randomisation, follow-up and outcomes assessment.

Patients are randomised either to receive the dietary modification and physical activity intervention or standard care (control group) with a 1:1 allocation ratio (Figure 1).

### Setting and participants

Prostate cancer patients are recruited from the Cancer Centre at Belfast City Hospital, Belfast, Northern Ireland, United Kingdom. Our inclusion and exclusion criteria are as follows.

### Inclusion criteria

1) histologically proven adenocarcinoma of prostate; and 2) commencing Lutenising Hormone Releasing Hormone Agonist (LHRHa) therapy for at least 6 months or already being treated with LHRHa and planned to continue for at least a further 6 months

### Exclusion criteria

1) co-morbid conditions that limit physical activity such as severe cardiac disease, recent myocardial infarction, severe asthma or breathlessness, uncontrolled hypertension (blood pressure > 160/95 mm/Hg), or severe pain; 2) medical conditions that require a reduced fruit and vegetable diet (e.g. kidney failure); 3) history of insulin dependent diabetes; 4) treated with any type of steroid hormone; 5) treated for any other cancer; and 6) life expectancy of less than 2 years.

### Sample size

This study is an exploratory one and so formal power calculations are difficult. The primary endpoint used to calculate sample size was body composition. Smith *et al *[17] presented data from a study of prostate cancer patients treated with hormone therapy and the mean percentage change in fat mass following ADT treatment was 9.4% (SD = 9.6%). If it is assumed that the controls in the planned study will have a similar percentage of fat mass, then with 36 patients in the intervention group and 36 patients in the control group the study will have 90% power at 5% significance to detect a mean percentage change in fat mass of 7.4% between the control and intervention group. An additional 30% was added to the sample size calculation to account for non-compliance and potential drop-out or discontinuation. Therefore 47 patients will be randomised to the intervention and 47 randomised to the control group. This sample size will also allow us to detect a 3.4 point (SD = 5.0 points) difference in Fatigue Severity Score [8] (a 3 point difference on the fatigue scale is considered to be the minimal clinically important difference) [18]; and a 6.0 point (SD = 9.0 points) difference on the Functional Assessment of Cancer Therapy-General (FACT-G) score [8].

### Recruitment of patients

Hospital notes are initially checked to identify eligible patients. The patient's consultant confirms their eligibility in terms of clinical conditions related with mobility. The study objectives, hypotheses, design and data collection procedures are then discussed with patients who meet the inclusion criteria. They are given an information sheet to read at home and are then phoned within 3-5 days to get their verbal consent to participate in the study. An appointment date is arranged for collection of baseline data at the clinic. Prior to the visit, a set of self-administered questionnaires are mailed to the patient for completion and they are asked to bring the completed questionnaires to their next visit. Patients provide written consent on the day of recruitment; before data collection. Midterm and final assessment dates are then scheduled for a 3 month (13 weeks) and 6 month (25 weeks) follow-up visit (Figure 1).

### Randomisation

Patients are randomised using a block randomisation approach with computer generated random numbers, to the intervention and control group, with equal probability that they are randomised to the intervention or control arm. Using a block size of four there are six sequence permutations to which we can allocate patients to the intervention (I) and control (C) arms: IICC, ICIC, ICCI, CIIC, CICI, and CCII. One of the six permutations was selected randomly and then four patients assigned accordingly. The process was repeated to allocate patients to the intervention and control arms for the required sample size. An independent researcher constructed the allocation sequence and sealed these in individual opaque envelopes with study identification numbers on the front. Patients are only numbered once they have provided verbal consent by phone. Randomisation takes place at an individual level after the baseline measurements have been taken and approximately one week prior to the start of the intervention. Therefore, the researcher does not know the allocation of the patients until the baseline data collection has been completed. Similarly patients are consented to the study before they know which group they will have been allocated to.

### Intervention

Those patients randomised to the intervention arm receive the intervention from a qualified nutritionist. Within a week after their baseline measurements are taken, the nutritionist visits the patients at their home to describe and deliver the intervention which has two components; dietary modification and physical activity.

#### Dietary modification

The dietary advice is developed based on the patients' usual dietary intake, measured at baseline using a 7-day food diary. They are provided with individually tailored advice to encourage them to adopt a diet that is commensurate with current dietary guidelines [19] and, specifically, to meet the following criteria;

a) Eat 5 or more servings of vegetables and fruits per day

b) Reduce total fat intake to 30%-35% of total energy, with < 10% of energy from saturated fat

c) Limit polyunsaturated fat intake to 10% of daily total energy intake

d) Limit consumption of processed meats

e) Eat fibre-rich foods. Aim to consume 25 to 35 g of fibre daily

f) Limit alcohol intake ≤ 28 units/week

g) Limit intake of foods high in salt and/or sugar

#### Moderate physical activity

Brisk walking at least 30 min per day, in addition to usual activities on 5 or more days of the week.

The diet and physical activity intervention is tailored to each patient based on the information collected during their baseline interview. The nutritionist meets each patient to discuss their calculated energy and nutrient requirements. Dietary recommendations are based on the information collected on weight, physical activity levels and baseline dietary intake. Energy is calculated based on actual body weight; however, 500 kcal/day reduction is advised to the overweight/obese patients to prevent or reduce weight gain. Each patient in the intervention arm receives an individualised guidebook with tips and guidelines to help adherence to the diet. The guidebook includes; a sample menu to provide an example of the amounts and type of foods that can be included for breakfast, lunch, dinner and snacks; a list of portion sizes for foods typically eaten; tips to incorporate more fruit, vegetables and fibre, and reduce total fat, saturated fat, sugar and salt; a list of healthier foods choices and a list of foods that should be chosen less frequently from all food groups; examples of healthy alternative menus for breakfast, snacks, lunch and dinner.

The physical activity intervention is also tailored to each patient based on their baseline physical activity level. As a minimum, each patient is asked to walk at a brisk pace for at least 30 minutes per day, 5 times or more per week. However, men who are already physically active are advised to increase the duration and frequency of walking each week. They are also asked to set a goal of increasing the number of steps by at least 10% every week. A pedometer is provided to each participant in an effort to encourage them to comply with the physical activity intervention and as an independent measure of physical activity levels during the intervention period.

Men in the control group will receive standard care during the intervention period but will be offered the same guidelines at the end of the 6 month intervention.

### Data collection

Data to evaluate the efficacy of the intervention are collected at baseline and at 3 and 6 months after the intervention has begun in the intervention and control group. Data are collected via interview, self administrated questionnaire, physical and biological measurements. Data collection instruments and the study timeline are summarised in Table 1. All instruments used have been previously validated and we also pre-tested them in the Northern Irish population prior to use in the study.

**Table 1:** Data collection instruments at different assessment points

	Baseline	3-month follow-up	6-month follow-up
Anthropometry	**X**	**X**	**X**

FSS	**X**	**X**	**X**

FACT-P	**X**	**X**	**X**

7-DPARQ	**X**	**X**	**X**

6-min Walk Test	**X**	**X**	**X**

PSS-10	**X**	**X**	**X**

7DD	**X**	**X**	**X**

Fasting blood sample	**X**	**X**	**X**

TTM	**X**		

Questionnaire for background information	**X**		

### Primary outcomes

The three main outcomes of interest are changes in body composition, fatigue and quality of life.

#### Body Composition

Measures of body composition include percentage body fat (assessed from skin fold thickness measured at 4 sites; triceps, biceps, subscapular and suprailiac using a Harpenden Skinfold Calipers), height, (measured to the nearest centimetre using SECA Leicester Height Measure), weight (SECA 704 Electronic Column Scale), body mass index (BMI) (Kg/M^2^), waist and hip circumference as a measure of central adiposity and mid upper arm circumference as a measure of lean body mass. A standard protocol is used to conduct all anthropometric measurements [20].

#### Fatigue

The Fatigue Severity Scale (FSS) is a self-reported scale and has been used previously in prostate cancer patients treated with ADT [21] and in patients with advanced stage cancer of the prostate [22]. Patients are asked to report their degree of agreement (on a seven-point scale) with nine items related to fatigue. Therefore, scores can range between 9 (indicating minimum fatigue) and 63 (indicating maximum fatigue). The validity of this scale is supported by its good correlation with both the EORTC Fatigue scale (Spearman Rank Correlation Coefficient r = 0.83) and the bi-dimensional fatigue scale (r = 0.62). A previous study of cancer related fatigue reported that 95% of an elderly control population without cancer scored less than 42 on the FSS scale [22]. Therefore, in this study 'severe fatigue' for cancer patients was defined as a score of 42 or greater based on the results of a study by Stone et al in prostate cancer patients treated with ADT [21].

#### Quality of life

The Functional Assessment of Cancer Therapy-Prostate (FACT-P) is used to assess prostate cancer related quality of life [23]. The FACT-P is a 40-item self-reported questionnaire assessing a range of quality of life domains: physical, social/family, emotional, and functional well being. In addition, a 12-item prostate cancer subscale (PSC) which is specific to the symptoms and side effects associated with prostate cancer treatment such as body image, pain, urinary and sexual functioning is also used. Responses to questions use a five-point Likert-type scale ranging from 0 (not at all) to 4 (very much). The scores are summed to produce a subscale score for each domain assessed. The subscale scores range from 0 to 28 for the physical, social/family, and functional well being scales, 0 to 24 for the emotional well being scale, and 0 to 48 for the PSC, and higher scores represent better quality of life. The scores of these four domains are summed to calculate the FACT-G (Functional Assessment of Cancer Therapy-General) and total scores of FACT-G and PSC are added up to produce a FACT-P score. An additional score, the Trial Outcome Index (TOI) is created by summing the physical well-being, functional well-being and PCS scores. This instrument has been used previously in prostate cancer patients receiving ADT [24]. It has been considered a reliable instrument with sensitivity to change in prostate specific antigen (PSA) scores and performance status [23].

### Secondary outcomes

Secondary outcomes include nutrient intake, physical activity and perceived stress.

#### Nutrient intake

To reduce the possibility of memory recall problems for this older patient group a 7-day food diary (7DD) is used to assess food and nutrient intake. The patients are asked to record, in as much detail as possible, all food and beverages consumed over a 7-day period. The food diary contains instructions for completion, one page to record foods and drinks eaten during seven time points (before breakfast, breakfast, before lunch, lunch, before dinner, dinner, after dinner) and alcohol over a 7 day period. The instructions included the provision of typical portion sizes to help the patients to indicate the amount of each food consumed. Patients are also encouraged to describe the portion size consumed using other measures if they wish, such as weight in grams or ounces, or in household units, such as tablespoons, cups, slices etc. At the time of the patients' visit the trained nutritionist clarified any omissions and collected additional information if required such as the cooking methods used, brands of foods consumed and portion sizes if these were unclear. The 7DD has been used in the UK EPIC (European Prospective Investigation of Cancer and Nutrition) study [25] amongst others and is a manageable method for our population. It has been demonstrated that a 7DD provides a better estimate of average intake than a food frequency questionnaire or a 24 hour-recall [25,26]. A computer based software programme (WISP) will be used to interpret the food intake data to provide nutrient and food group intake estimates. The total number of servings of fruit and vegetable consumed will be calculated using the Food Standard Agency guidelines [19].

A fasting blood sample is collected for analysis of serum ferritin, antioxidant vitamins, dietary lipids (total cholesterol, high-density lipoprotein, low-density lipoprotein, triglycerides), C-reactive protein and albumin. A total of 30 ml of blood is collected and following appropriate processing for serum and plasma are stored in -80°C freezer.

#### Physical activity

Physical activity is assessed with 7-Day Physical Activity Recall (7-DPARQ) [27,28]. This is an interviewer-administered measure assessing current and recent past physical activity from the last 7 days. Information on moderate, hard and very hard activities and time spent sleeping is gathered from the interview and light activities is imputed from the time remaining. Additionally, patients report how typical the week's activity is compared to their activity in the previous three months. The 7-DPARQ measures energy expenditure and yields a summery score in kcal/kg^-1^/day^-1 ^for the previous week. It has been validated and used for prostate cancer patients in previous studies [12,29].

Functional capacity is measured using a 6-min Walk Test [30,31], which was used in previous studies of prostate cancer patients [9,29]. This test includes a measure of the distance walked, to the nearest metre in 6 minutes. This functional measure was chosen rather than a measure of physiological fitness as it appears to be a more suitable outcome for an elderly population [29]. The 6-min walk test has a high test-retest reliability over a 2-week time frame (*r *= 0.87) [32].

#### Stress

Psychological stress is assessed using the Perceived Stress Scale (PSS-10), which is the most widely used psychological instrument for measuring the perception of stress among prostate cancer survivors [33-35]. The PSS-10 is a 10-item questionnaire designed to measure the perceived stress and the degree to which patients found their lives unpredictable, uncontrollable, or overloaded in last 1 month. Questions are rated on a 4-point Likert scale with responses ranging from 0 = "never" to 4 = "very often." [36]

#### Other assessments

The transtheoretical model (TTM) is used to assess a patients' readiness to change their lifestyle behaviour [37]. Patients will be categorised according to their stage of readiness to change and this information will be taken into consideration in the analysis and interpretation of the impact of the intervention. Patients are asked, 'on average do you - eat at least 5 servings of vegetables and fruits each day; avoid eating too much fat; walk 30 minutes every day at a brisk pace, 3 to 5 times/week?'. Responses include: Yes - 'more than six months' (interpreted as maintenance), 'less than six months' (interpreted as action); No - 'intend to in the next 30 days' (interpreted as preparation), 'intend to in the next 6 months' (interpreted as contemplation) and not intending to in the next 6 months (interpreted as pre-contemplation). Stage of readiness to change is also considered in the delivery of the intervention. For example, the potential benefit of a dietary and physical activity intervention is really emphasised to those men who are in a stage of contemplation/precontemplation with respect to making diet and physical activity changes.

A structured questionnaire is used to collect information on age, socioeconomic status, education, family and social support, tobacco and alcohol intake, barriers to exercise and diet, supplementation use and family history of prostate cancer. A clinical history, including the date of diagnosis, stage of disease and treatment details is recorded from the patient's medical notes during screening. Data related to adverse events are collected continuously via follow-up phone call.

### Quality control

Following the initial visit, patients are contacted by phone every 2 weeks for the first 3 months and every 3 weeks thereafter to monitor progress and compliance with the intervention and to record any possible adverse events. The control group is also contacted every 6 weeks, for a general discussion about their condition. To standardise the follow-up call a written check-list has been used and relevant information (walking steps from the previous week and their goal for the current week, illness etc.) are collected. Each participant is encouraged to bring their partner or care-giver on the day of data collection and to be present for the nutritionist's visit at their home to help to answer questions regarding dietary intake and food preparation for those who may not be actively involved in food shopping and cooking. It is also hoped that by involving the patient's partner/care-giver, the patient is encouraged to comply with the intervention at home. The baseline and follow-up (3 month and 6 month) body composition measurements are completed by the same person to avoid inter-interviewer bias. However, this person is not masked to treatment assignment. Although it is preferable to have any subjective measurements, such as body composition measurements, taken by a person who is masked to treatment allocation, this was not possible in the present study and in order to minimise any bias that may be introduced, all body measurements are taken and recorded without referring to the previously recorded measurements.

### Compliance and drop-outs

Compliance is monitored through follow-up telephone calls and will be reported. Adherence to the intervention is examined at the patient's 3 month visit and an additional session with the nutritionist is arranged if necessary to provide additional dietary advice, recalculation of energy requirements and for encouragement. This also provides an opportunity to address any problems or difficulties being encountered by the patient, and to provide feedback. The number of patients that drop-out and their reasons for doing so will be documented but following the intent to treat analysis they will be included in data analysis until the point of drop-out. Differences in dropout rates and the reason for drop out will be assessed by group (intervention versus control).

### Data storage and confidentiality

All questionnaires are stored in a locked cabinet in a locked room, and have a unique identification number. Consent forms are stored separately from study questionnaires in a locked cabinet. Only anonymised data is entered into the computerised study database, and access to the database is restricted to the study team. Data will be stored for 17 years according to rules of the Belfast Health and Social Care Trust.

### Statistics

The data collected in the trial will be analysed based on intention to treat and lost to follow-up cases will be considered as missing observations [38]. Change over the treatment period will be calculated by subtracting the baseline value from the value at month 3 and month 6. Primary and secondary endpoints between those assigned to the intervention and control group will be compared using analysis of covariance (ANCOVA) with baseline scores included in the models as covariates. Skewed data will be log transformed prior to inclusion in the ANCOVA models. The trial outline conforms to the Consort statement guidelines.

### Ethical considerations

The study protocol has been approved by the Office for Research Ethics Committees Northern Ireland (ORECNI), the Research Governance of Belfast Health and Social Care Trust and Queen's University Belfast. Permission to contact patients is received from the consultants of the Northern Ireland Cancer Centre before approaching the patients. Informed written consent is collected from each participant. A telephone number and contact address is provided to patients for any queries they may have during the study period.

## Discussion

As far as we are aware, this is the first RCT to assess whether a combined diet and physical activity intervention can attenuate the negative changes in body composition associated with use of ADT in prostate cancer patients and also to assess its impact on fatigue and quality of life. The intervention period in the present study is longer than previous studies (12-20 weeks of physical activity interventions [8-11]) and, in keeping with current views that initiation and maintenance of a diet and physical activity modification for cancer survivors should be tailored to the survivor's condition and personal preferences [16], it is individually tailored to the patient. Moreover, patients' readiness to change lifestyle behaviours using the transtheoretical model is considered during the delivery of the intervention. This model has been used successfully in previous diet and exercise intervention studies [12,13,39]. We expect that by using these approaches we will increase compliance and achieve the greatest possible change in behaviour and in the outcomes of interest. The intervention is also home rather than hospital based and is therefore more likely to be sustained in this older population group [13,14,40,41]. Moreover, the study sample size is sufficient to detect a clinically relevant change in body composition and the study includes a range of validated physiological and psychological assessments, which will maximise the internal validity of the study.

The majority of ADT treated prostate cancer patients are overweight and obese [42]. Though we are not promoting a weight reducing diet, and weight reduction is not the primary goal of the intervention, it is likely that an improvement in dietary intake and a higher level of physical activity will result in modest weight loss in the intervention group. It is known that overweight and obesity is associated with an increased risk of chronic diseases including cardiovascular disease, diabetes, some forms of cancer and recurrence of cancer. Therefore, maintenance of weight or gradual weight loss in the intervention group can be viewed as a favourable outcome [43]. Because our intervention incorporates both dietary modification and physical activity, we will not be able to separate out the effects specifically attributable to either of these components. Furthermore, with no allocation concealment the control group patients will be free to change their diet and increase their physical activity; even though such behaviour may weaken any observed associations in the intervention arm.

The external validity of the study will depend on the representative of the patient population from which we recruit and on the proportion of eligible patients who agree to participate in the study. For logistic reasons, recruitment is limited to one centre, which is the regional oncology centre (in Belfast) and patients treated within this centre may not be representative of all prostate cancer patients treated with ADT. Also, the characteristics of recruited patients may be influenced by the study design e.g. patients who agree to take part in the trial may be more likely to be older or retired or highly motivated, as involvement in the study demands three additional clinic visits for outcome measurement. Patients living outside of the Belfast area may be more likely to decline in order to avoid more frequent journeys to the clinic and the associated additional costs that may be incurred. However, we are making every effort to arrange our follow-up visits to coincide with the patients' 3-monthly follow-up visits with their consultants to minimise travel. A positive aspect of our approach to recruitment is that potential participants have to be initially approached by their consultant which may lead to a better uptake than other approaches to recruitment e.g. community advertisement [44].

Thus study will determine whether a healthy life style intervention in prostate cancer patients can attenuate the adverse body composition changes associated with ADT. If successful, this intervention may lead to substantial and important changes in the management of prostate cancer patients.

## Competing interests

The authors declare that they have no competing interests.

## Authors' contributions

FH, MMC, LJM, JMO were responsible for the study design. MMC is the principle investigator and conceived the project design. JMO facilitates patient recruitment in the clinical setting. FH and RFO are responsible for the coordination of patient recruitment and data collection and data entry. MMC, FH and RFO developed the intervention and RFO is responsible for delivery of the intervention. FH and RFO are responsible for the statistical analyses and all authors are responsible for interpretation of the results. FH wrote the first draft of the manuscript. All authors read and approved the final version of the manuscript.
